# Supplementary figures and images for: DRAMS: A tool to detect and re-align mixed-up samples for integrative studies of multi-omics data
Source: PLoS Comput Biol. 2020 Apr 13;16(4):e1007522. doi: 10.1371/journal.pcbi.1007522 (PMC7179940; doi:10.1371/journal.pcbi.1007522)

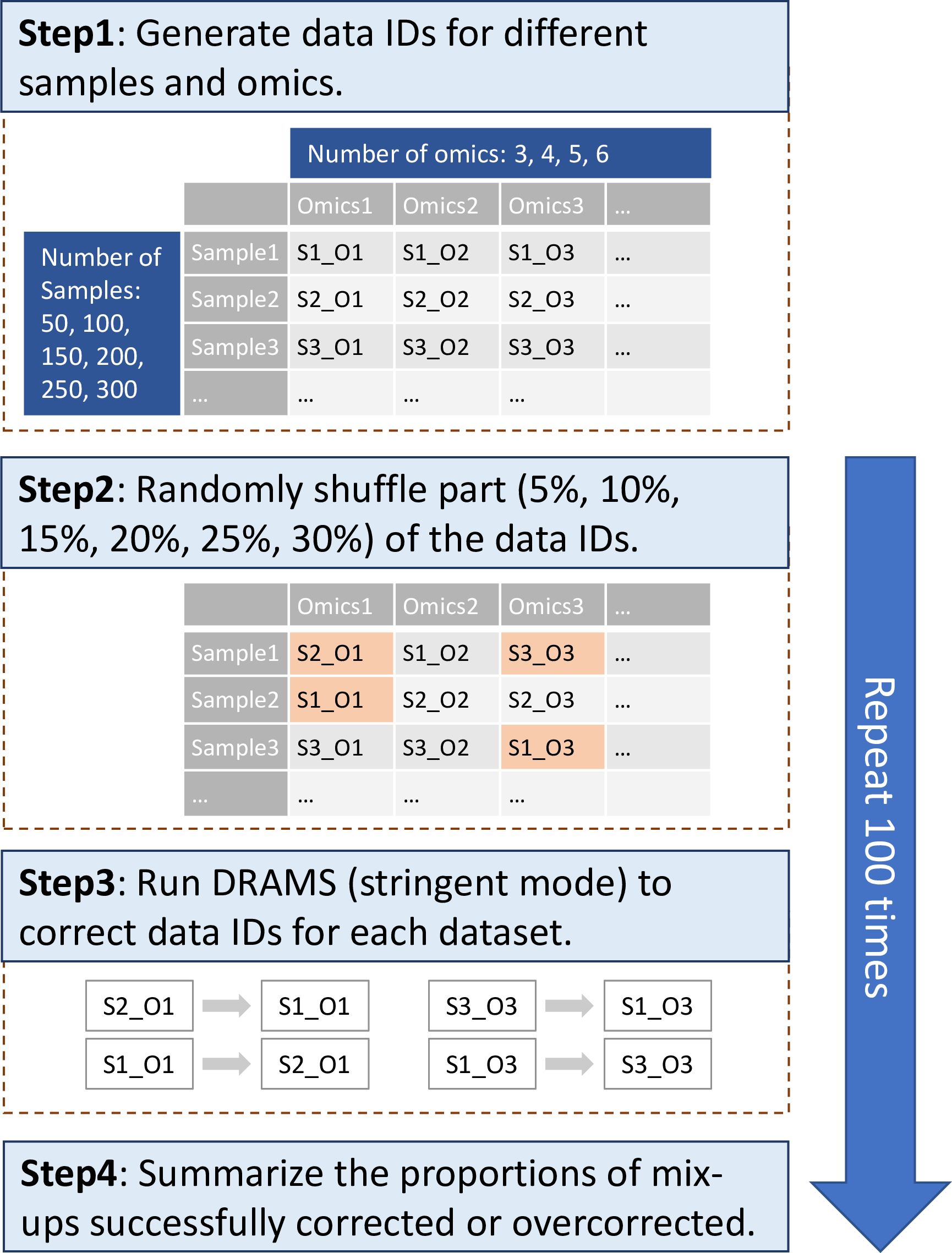

Supplement: S1 Fig — Simulation data were generated to test the performance of DRAMS. Step 2 to step 4 were repeated four times. (TIF) [file pcbi.1007522.s001.tif]

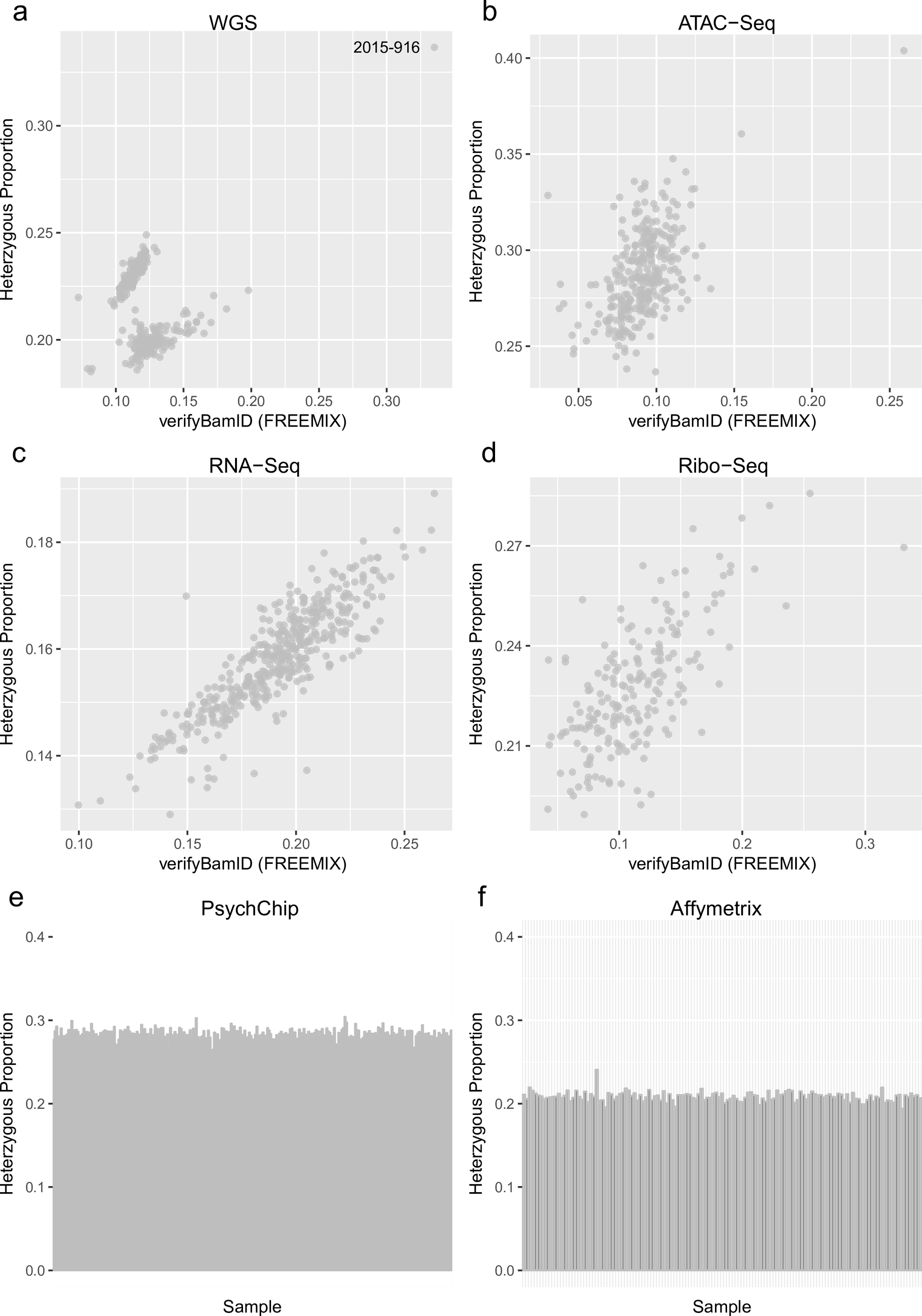

Supplement: S2 Fig — Two methods (Heterozygous proportion and VerifyBamID) were used to estimate sample contamination for WGS, ATAC-Seq, RNA-Seq, and Ribo-Seq data. For VerifyBamID results, “FREEMIX” (0–1 scale) was used to indicate possible sample contamination. For PsychChip and Affymetrix samples, we calculated only heterozygous proportions. (TIF) [file pcbi.1007522.s002.tif]

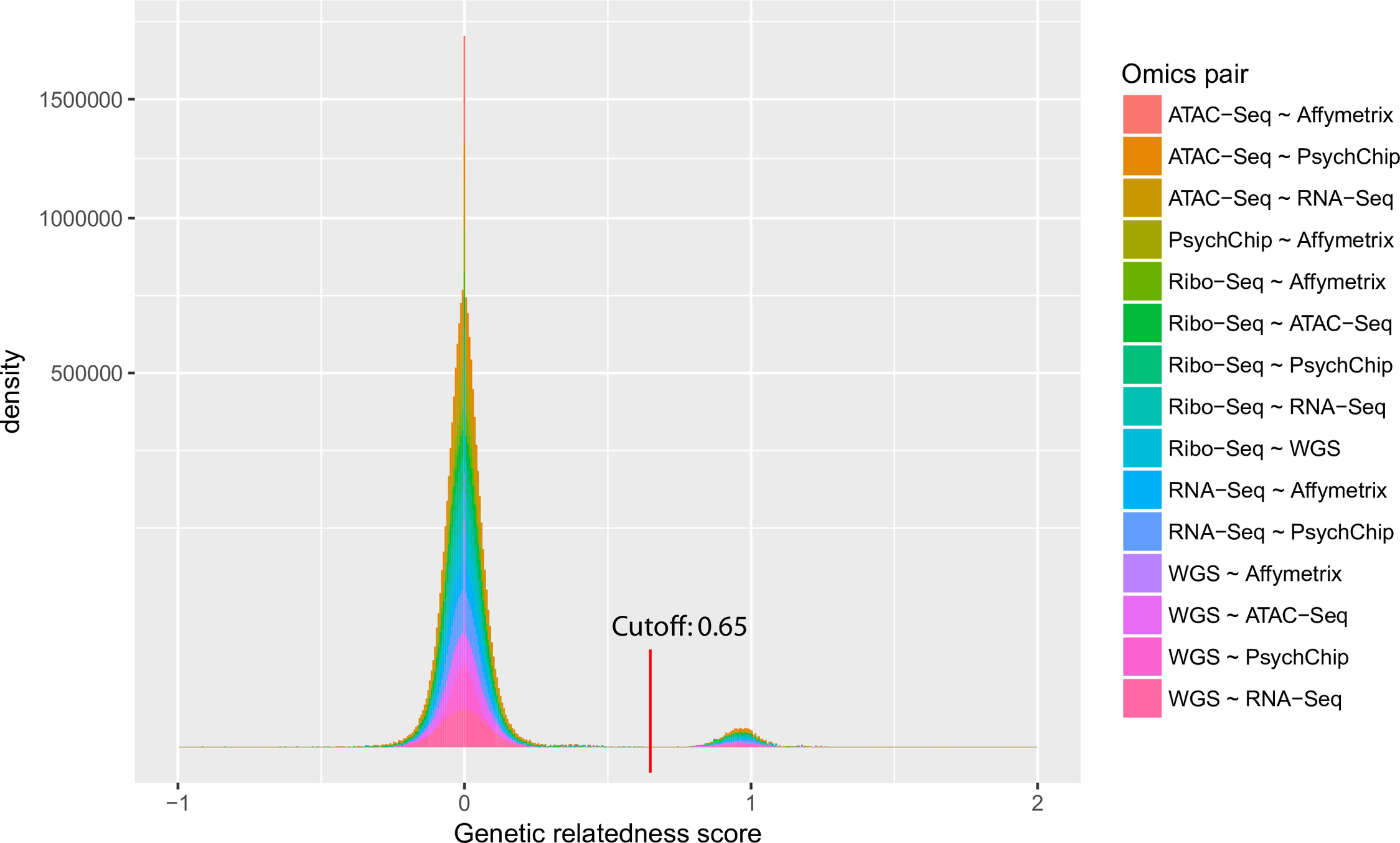

Supplement: S3 Fig — Genetic relatedness scores were calculated by GCTA. (TIF) [file pcbi.1007522.s003.tif]

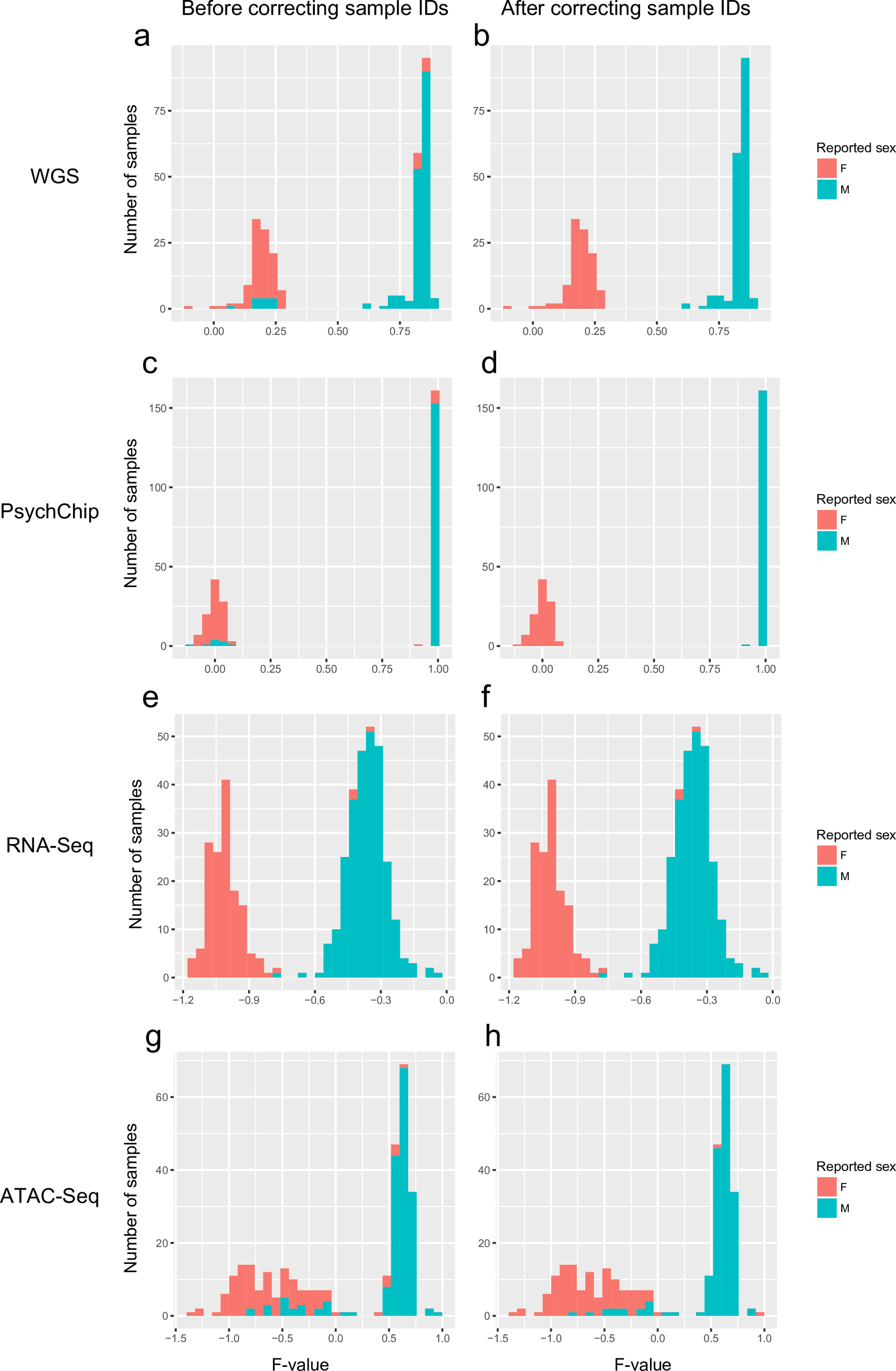

Supplement: S4 Fig — Genetics-based sexes were inferred using Plink. Larger F-value indicated that the sample is more likely to be male. Ribo-Seq and Affymetrix samples were not shown since we were not able to infer the genetics-based sexes. (TIF) [file pcbi.1007522.s004.tif]

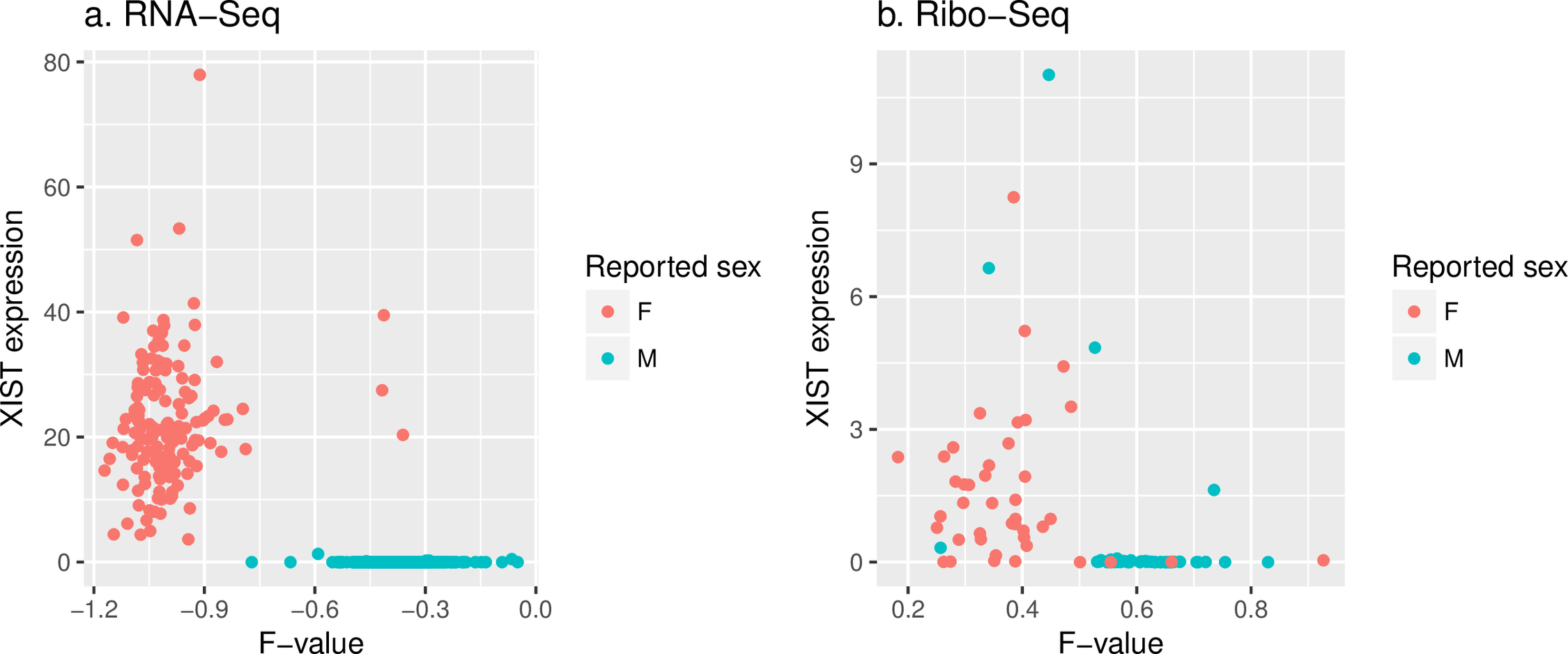

Supplement: S5 Fig — For sex chromosomes-inferred sexes, larger F-value indicates that the sample is more likely to be male. For XIST expression-inferred sex, the samples with XIST expression larger than zero are more likely to be female. The reported sexes were based on samples before ID correction. (TIF) [file pcbi.1007522.s005.tif]

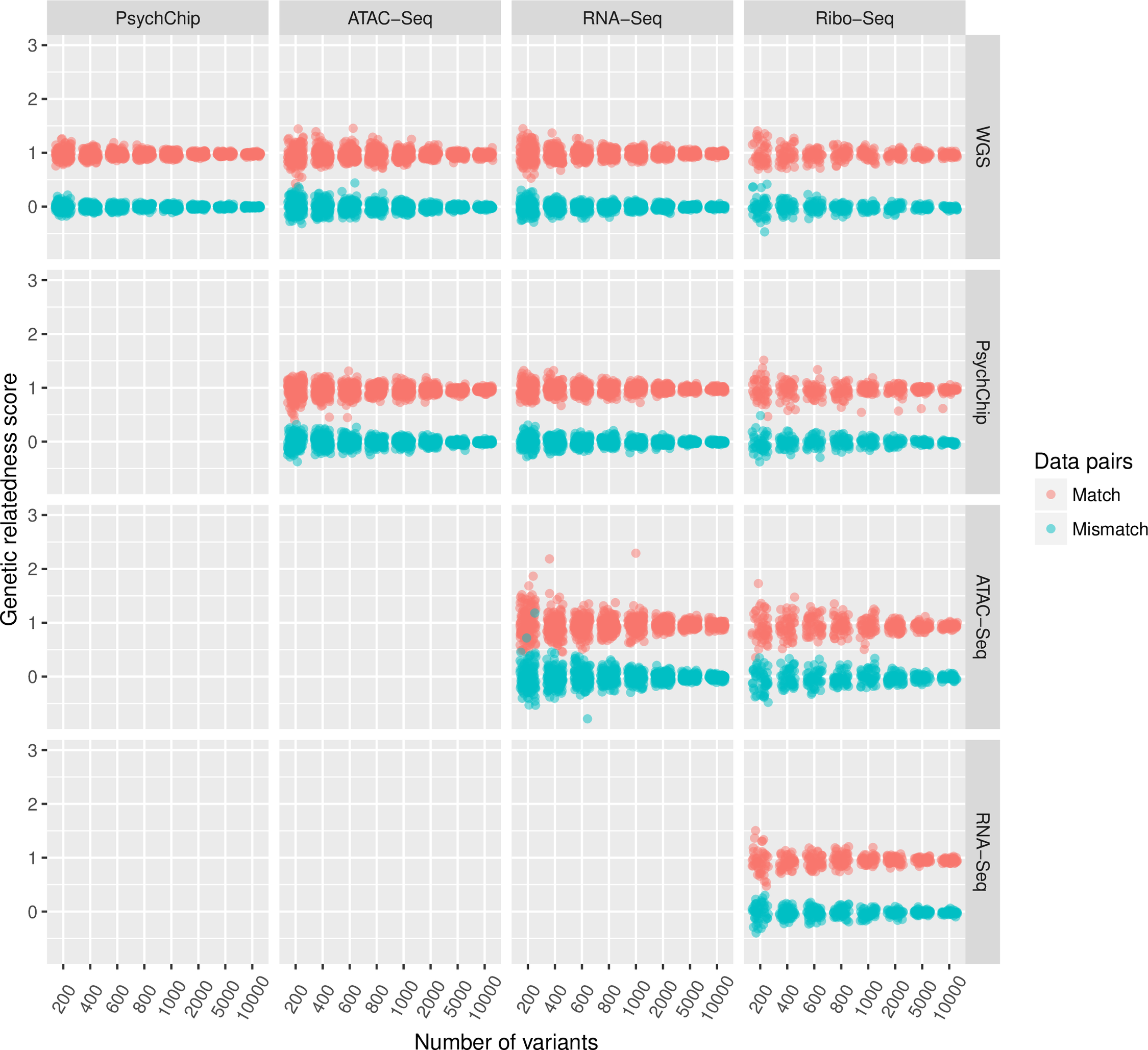

Supplement: S6 Fig — Genetic relatedness scores were calculated based on common SNPs (MAF>0.1) randomly selected from BrainGVEX data. Only the samples that matched well among all omics types were used. We only showed a subset of randomly selected mismatched data pairs according to the number of matched data pairs. (TIF) [file pcbi.1007522.s006.tif]

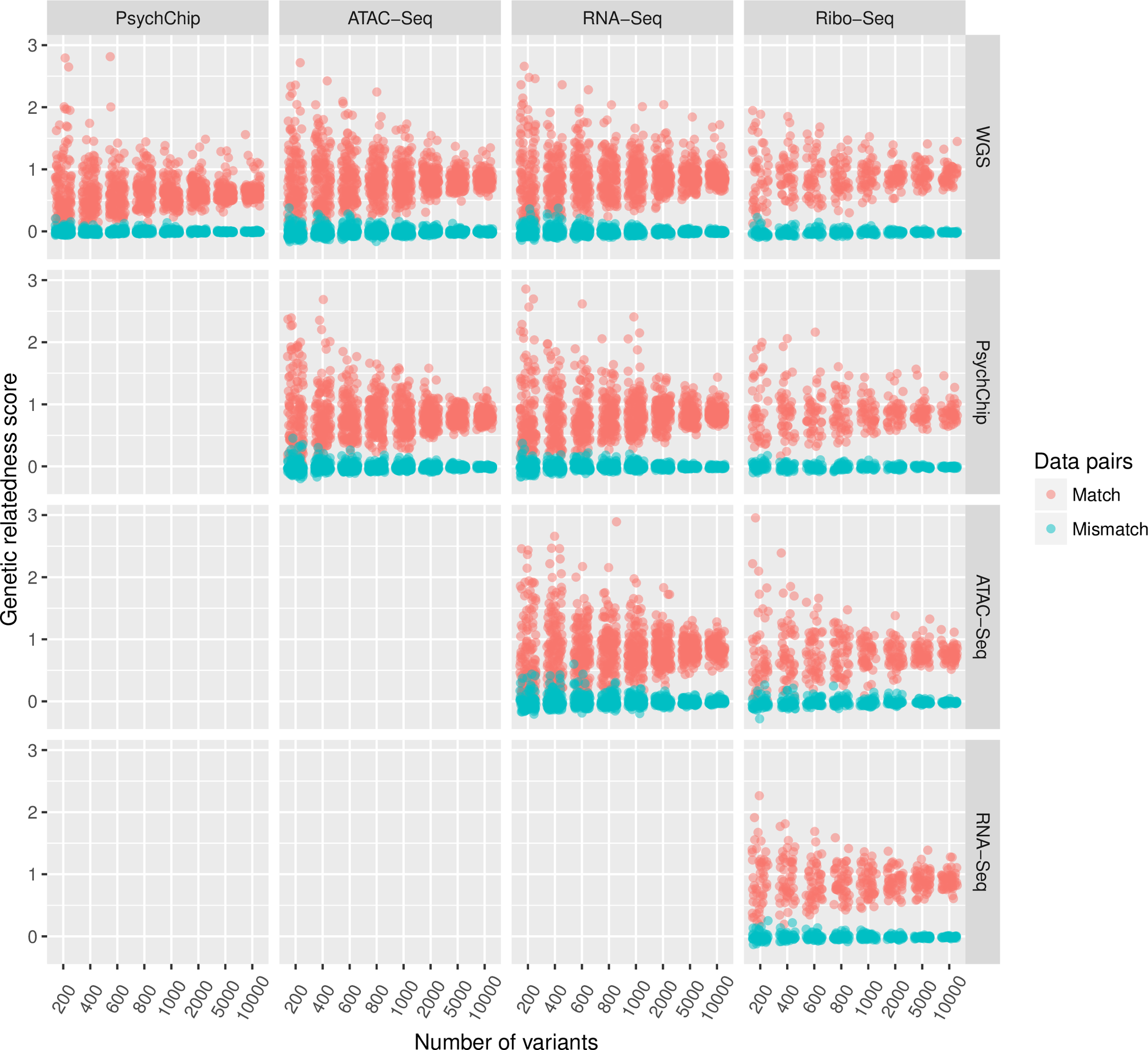

Supplement: S7 Fig — Genetic relatedness scores were calculated based on randomly selected rare SNVs (MAF<0.1) from BrainGVEX data. Only the samples that matched well among all omics types were used. We only showed a subset of randomly selected mismatched data pairs according to the number of matched data pairs. (TIF) [file pcbi.1007522.s007.tif]

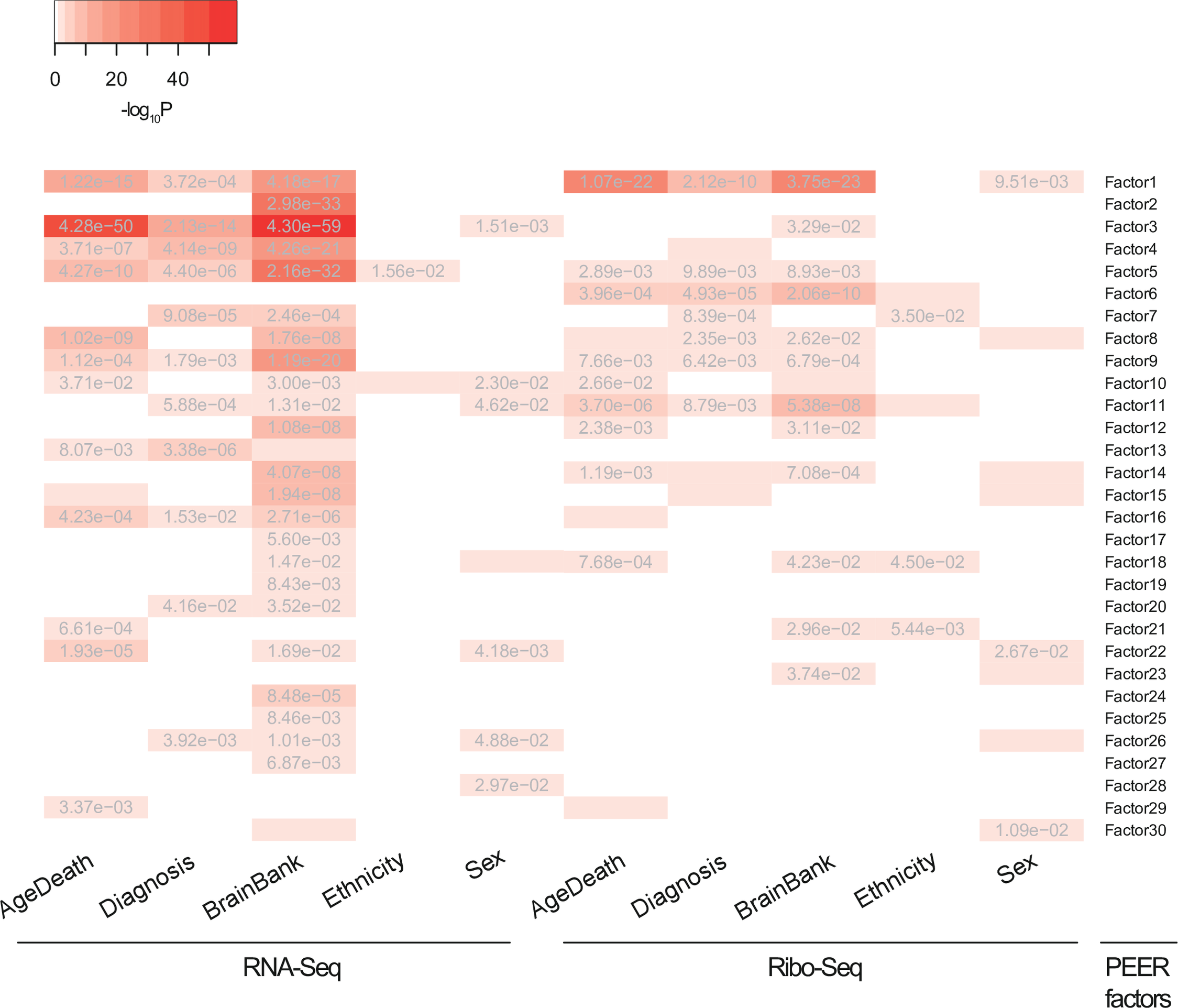

Supplement: S8 Fig — Spearman correlation tests were performed between PEER factors and ageDeath. One-way ANOVA tests were performed between PEER factors and Diagnosis, BrainBank, Ethnicity, and Sex. P values were marked for the cells with significant correlation (P value < 0.05). (TIF) [file pcbi.1007522.s008.tif]
