# Supplementary material for: DRAMS: A tool to detect and re-align mixed-up samples for integrative studies of multi-omics data
Source: PLoS Comput Biol. 2020 Apr 13;16(4):e1007522. doi: 10.1371/journal.pcbi.1007522 (PMC7179940; doi:10.1371/journal.pcbi.1007522)
Supplement: S1 Text — (DOCX) [file pcbi.1007522.s018.docx]

**Comparison of GCTA and NGSCheckMate in estimating genetic relatedness**

We compared the two tools, GCTA and NGSCheckMate, in estimating genetic relatedness and extracting highly related data pairs using our BrainGVEX data (S9 Table). GCTA estimates genetic relatedness by calculating genotype concordance rates which also takes Allele Frequencies (AFs) into consideration. NGSCheckMate estimates genetic relatedness by calculating the correlation of Variant Allele Fractions (VAFs) between every two samples based on a fixed set of SNPs. We found that, although the two tools estimated genetic relatedness in different ways, they have large concordance rate for most of the -omics types (average concordance rate for matched sample pairs: 99.6%; average concordance rate for mismatched sample pairs: 92.8%). For genetic relatedness between data from ATAC-Seq and Ribo-Seq, the two tools showed less consistency, this might be due to the relatively small overlapped region between the two -omics types.

Generally, GCTA can be used for both high-coverage sequencing and SNP array data. NGSCheckMate is specifically designed for low-coverage sequencing data, but might not be accurate for target sequencing data given that it only captures a subset of SNPs. In this study, given that NGSCheckMate is not applicable to PsychChip and Affymetrix data, we run DRAMS for BrainGVEX data based on the GCTA results.
