# Supplementary material for: DRAMS: A tool to detect and re-align mixed-up samples for integrative studies of multi-omics data
Source: PLoS Comput Biol. 2020 Apr 13;16(4):e1007522. doi: 10.1371/journal.pcbi.1007522 (PMC7179940; doi:10.1371/journal.pcbi.1007522)
Supplement: Attachment — Submitted filename: Responce to reviewers.docx [file pcbi.1007522.s019.docx]

Reviewer #1: Jiang et al. developed a tool to detect and correct mixed-up samples in multi-omics data. The mix-up refers to switched IDs during the data generation process. Through analyzing the PsychENCODE BrainGVEX data, they corrected 12.5% mixed-up IDs, and this correction is shown to improve the discovery of cis-QTL. I think the paper addressed an important and interesting research question, and it was written in a convincing way. Below I list some comments that may improve the manuscript.

Major comments:

“We can cluster all the highly related data and consider that the data from one cluster have only one potential ID.”
It was not sure how the clustering was done. Is it based on a clustering algorithm? What is the resulted cluster size? Is it restricted to be the number of data types? Is it necessary that each cluster has each data type? More details are expected to explain the clustering procedure.
A: Thanks for bringing up this question. Instead of “cluster”, we think that “group” should be more suitable in our manuscript, as there is no clustering algorithm in the model. Therefore, we changed the word “cluster” to “group” throughout the manuscript. We defined a “group” as a set of data, in which each data was highly related to at least one of the other data based on the genetic relatedness scores.

The size of a group is influenced by the number of omics types. We used six omics types in our PsychENCODE data. The group size in our dataset is mostly ranged from three to six. It’s not necessary that the group size is equal to the number of omics types, as we may not produce data for all omics types for each sample. For this situation, some groups may only include a subset of the omics types. We revised the manuscript at line 128-129 and 347-349.

In Fig 1 and S2, the range of genetic relatedness score exceeds 1. Is it really possible to exceed one or just because of density curve smoothing? If the latter, the authors may consider using a way to estimate the density within a restricted range.
Additionally, in Fig 1, “The text in each node represents the data ID. Different colors represent different omics types.” The pair of A-A in Step 1 Type 1 are in the same color. Should A-A be in different colors?

A: Yes, it’s possible to exceed one. The genetic relatedness scores were calculated using the GCTA software (2011 Yang et al, Am. J. Hum. Genet.). As shown in the formula below, ‘*x*’ denotes dosage genotype ranging from 0 to 2, ‘*p*’ denotes allele frequency. For the subscripts, ‘*i*’ denotes the index of SNPs, ‘*j*’ and ‘*k*’ denote the index of samples. Based on the formula, we can see that although the expected value is in the range of [-1, 1], the range of an actual value based on the data is not necessarily ranging from -1 to 1.

For the second question, we appreciate that the reviewer pointed out this mistake. A-A should be in different colors. We’ve changed the color in the new version.

In Fig 2, the authors assessed the proportions of successfully corrected mix-ups. I am wondering if there are non-mix-ups are wrongly classified as mix-ups and then overcorrected. For example, if using the cutoff of 0.65 of genetic relatedness scores for highly related data pairs, are there any non-mix-ups wrongly classified as mismatched data pairs? It may be related to the section of "Sensitivity and specificity in extracting highly related data pairs," but they are in different simulation settings.
In the simulation, how are the different data types simulated? How is the logistic regression fitted? Is it the same fitted model based on 44 data pairs as in the real data analysis?
The overcorrection may be an issue in the analysis of PsychENCODE data. “We identified a total of 1971 matched pairs and 518 mismatched pairs… In the end, we corrected 201 (12.5%) IDs for data of the six omics types.” There percentage of mismatched pairs and corrected IDs seems high.
A: Thanks for the comment. It’s definitely important for us to avoid over-correction. As you suggested, for the results of simulation data, we added the proportions of overcorrected mix-ups in Figure 2 (grey violin plots) and Supplementary table 3. From the figure and table, we can see that only a very small proportion of data were overcorrected. Noted that we used the stringent mode of DRAMS to correct mix-ups in simulation data. In the stringent mode, we discarded the data groups with less than three data or with no shared IDs (i.e. all data IDs in a group are different), as they are almost unlikely to be corrected.

For the simulation process, we’re sorry about the unclear description. We have added a figure (S1 Fig) to explain the simulation process and revised the manuscript at line 545-562 (“Simulation data” in “Materials and Methods”). Briefly, we generated data IDs for different samples and omics and randomly shuffled parts of the data IDs to simulate mix-ups. Then, we used DRAMS to correct the data IDs and calculated the proportion of corrected and over-corrected items for each simulation. The parameters trained by the PsychENCODE BrainGVEX data (the 44 data pairs) was used to run DRAMS in simulation data.

For real data, we observed no evidence for data overcorrection. But we have several evidence that most of the data were successfully corrected. After correcting data IDs, we mapped more QTLs, and comparing with the discarded eQTLs, the novel eQTLs preserved more in GTEx (Table 2). After correcting data IDs in the PsychENCODE data, we successfully matched races, especially for all those cross-races mix-ups. This is a strong evidence that the data IDs were correctly aligned.

Minor comments:

Fig 4 has a lot of abbreviations. Providing the full names in the caption would help reading.

A: Thank the reviewer’s suggestion. We added full names in the captions.

The Supplementary Figures need captions.

A: Thank the reviewer’s suggestion. We added captions for Supplementary Figures at the end of the manuscript.

“Since we intend to test whether the number of cis-QTLs increased, only chromosome 1 was used to save computing time.” This should be at least noted in the caption of Table 2 to help interpret the number of eQTLs.

A: Thank the reviewer’s suggestion. We have revised the caption as suggested.

Page 19, “In formula 3, Sa and Sb indicate the sex matching level for data “A” and “B”, respectively. Taking Sa as an example, **the original value of Sa would be 0**. If the reported sex and genetics-based sex in data “A” are matched, Sa would be **plus 0.5**. If the reported sex of data “A” and the genetics-based sex of data “B” are matched, which means that the reported sex and genetics-based sex are matched after switch ID from “B” to “A”, then Sa would be **plus 0.5**.” Could you explain more why the values of Sa are set in this way? It seems hard to understand and confusing.

A: We’re sorry about the confusion. We’ve revised this paragraph at line 505-509 as suggested. Here is a copy of the revised sentences:

**In formula 3, Sa and Sb indicate the sex matching level for data “A” and “B”, respectively. Taking Sa as an example, it indicates 1) whether the reported sex and genetics-based sex in data “A” are matched, and 2) if assigning ID “A” to data “B”, do the reported sex of data “A” and the genetics-based sex of data “B” match? We assign a score 0.5 for each of the two conditions.**

Grammar error. Page 2: “As the number of datasets to be integrated increaseS”
Page 3: “all omics data of the same samples were clusterED together”
Page 4: “For the first step is to build highly related data pairs.”
Page 18: “those pairS with smaller scores were classified”

A: Thanks so much for kindly reminding us the grammar error. We have corrected all the errors.

Reviewer #2: Comments to authors
The manuscript by Yi and colleagues, “DRAMS: A Tool to Detect and Re-Align Mixed-up Samples for Integrative Studies of Multi-omics Data”, presents a new method to detect and re-align mixed-up samples in multi-omics studies. The authors calibrated DRAMS using simulations and applied their method to the data from PsychENCODE BrainGVEX project to correct 201 sample IDs. They further tried to validated the results by comparing PCA and eQTL results before and after correcting sample IDs. The experiment is well designed and the analyses were carefully conducted. As the increasing emergency of multi-omics data, this method would be useful.

Major comments:

1. The authors should compare DRAMS with state-of-the-art methods of this kind in both simulation and real data analyses, such as MixupMapper.

A: Thanks for the comment. Existing tools do have something similarity to our tool DRAMS, but they have different purposes. As we mentioned in the manuscript, the sample realignment procedure was comprised of two steps: first, to estimate genetic relatedness among the data of different omics and group all the data of the same individual; then, to assign potential IDs for each group. Existing tools, such as MixupMapper, MODMatcher, NGSCheckMate, GCTA, focused on the first step. They only matched data of the same origin and grouped the data together. However, after grouping, they didn’t propose a systematic solution on how to identify the potential true IDs for the mislabeled data. DRAMS is a brand-new tool to address a problem that will be more significant in the future. We feel we are not able to find a proper way to do a direct comparison with existing algorithms given these fundamental differences. We have highlighted the differences in INTRODUCTION at line 69-76.

2. DRAMS needs a set of hand-picked high-confidence mismatched data pairs as a training set. How many pairs with certain switch directions are required in the training process? What if there are no such mismatched data pairs?

A: We appreciated the reviewer’s suggestion. We do provide the hand-picked mismatched pairs (https://github.com/Yi-Jiang/DRAMS/tree/master/training). We’ve used 44 hand-picked high-confidence mismatched data pairs from the PsychENCODE data as a training set and trained the logistic regression model. The trained model was integrated in our scripts and set as default. Users can freely use our trained model or build their own model using their inhouse data.

It is free for users to train the model using their own dataset, as we’ve provided an option for users to train a model. If there are not enough mismatched pairs in the data, it does pose more challenges in training a model. But on the other hand, it means the mix-up is not a big problem and therefore we can just manually correct those few mix-ups.

3. The authors used a relatedness score of 0.65 to define highly related data pairs. Is it possible that the related individual pairs (due to relatedness rather than the same individual in different data types) have the relatedness score > 0.65? More justification is required here.
A: Thanks for bringing up this question. If related individuals exist, it is possible that the related individual pairs have the relatedness score > 0.65. For this situation, we recommend to use a stringent threshold to extract highly related data pairs using GCTA or NGSCheckMate. For GCTA, we recommend to eyeball the distribution of genetic relatedness scores and choose a higher threshold. For NGSCheckMate, the tool provided a “-f” parameter that define highly related sample pairs in a stringent mode, which will reduce the probability of mislabeling among relatives. However, even in the stringent mode, it is still possible that some related individuals be identified as the same individual. In other words, DRAMS need to be used with great caution when processing data from family members. That will be a major challenge for the algorithm, particularly on data with less usable genotypes or of more noise.

In the revised manuscript, we have added one paragraph in Discussion at line 376-385.

4. The authors claimed that more types of omics data were included, the power is higher. However, it would increase the computational complexity. In this case, how about the computational burden? It would be useful to summary the resources requirement (e.g., running time, memory usage) under different simulation scenarios.

A: We appreciate the reviewer for pointing out the computational burden issue. We summarized the computational timing for each step and added a section in “Materials and Methods” at line 594-601. Here is a copy of the contents:

Step 1, map reads to reference genome (FASTQ to BAM, for one sample, 4Gb data size): 4 CPU, 15G memory, 1.5 hours.

Step 2, call genotypes (BAM to VCF, for one sample, 4Gb data size): 12 CPU, 20G memory, 7 hours.

Step 3, infer genetics-based sexes (for one sample): 1 CPU, 1G memory, 1 minutes.

Step 4, calculate genetic relatedness scores (200 samples, 6 omics types): 1 CPU, 1G memory, 10 minutes.

Step 5, correct data IDs (200 samples, 6 omics types): 1 CPU, 1G memory, 5 minutes.

5. In the eQTL analysis, did the authors correct for ancestry and sex? In addition, as discussed by the authors, large number of QTLs does not necessarily mean correct data ID. It would be useful to replicate the new discovery of eQTLs in an independent data set.

A: For both RNA-Seq and Ribo-Seq data, we corrected 30 hidden factors estimated by PEER (Stegle et al. Nature Protocol 2012). We didn’t include ancestry and sex in QTL analyses, as these covariates were associated with one or multiple PEER factors. I added a figure to show the correlation between several known covariates and PEER factors (S8 Fig). As most of our samples are Caucasion (417 out of 426), we can only see a weak correlation (but still significant) between ancestry and PEER factors. We believe using PEER algorithm can remove the ancestry and sex effects completely.

As the reviewer suggested, we tested the proportion of novel and discarded eQTLs replicated in GTEx (Table 2). We can see from the table that ~50% of the novel eQTLs can be replicated in GTEx (denoted by π_1_). For the discarded eQTLs, only ~20% can be replicated.

Minor comments:

1. Figure 1 is quite busy. It is difficult for the readers to follow. Detailed legend is needed.

A: Thanks. We have revised the caption with more details at line 103-123.

2. Is it a typo of “404” mismatched data pairs, given 518 mismatched pairs in total and 44 pairs with certain switch directions?

A: Thanks for reminding us. It’s a typo. It should be “474” here. We have corrected it on the revised version.

3. In Table S4, there were 80 data pairs with the same ID were defined as “mismatched pairs”. It is not clear of the definition of “matched pairs” and “mismatched pairs” in Fig. 3. The authors also mentioned that eight data were not related to any other data. It does not make sense that only eight data are not related to any other data among all samples.

A: We are sorry about the mistake and we appreciate the reviewer for pointing out this. The Ribo-Seq IDs for the 80 mismatched data pairs were changed by mistake. This is because we temporally changed the Ribo-Seq IDs when doing a manual check but forgot to change back the original IDs in this table. Now we changed the Ribo-Seq IDs back. This does not have any influences on the subsequent results.

The “related data pair” was defined based on the genetic relatedness score estimated by GCTA. Based on the distribution of genetic relatedness scores (S3 Fig), we defined a data pair with genetic relatedness score > 0.65 as a highly related data pair. The words “matched pairs” and “mismatched pairs” were defined in the manuscript at line 130-133. Matched pairs: highly related data pairs that have the same individual IDs. Mismatched pairs: highly related data pairs with different individual IDs. To make it easier to follow, we also added this annotation in the caption of Fig. 3.

We produced six omics types for our samples. For most of the samples, data of at least two omics types were produced (S1 table). Therefore, it is reasonable that we only have eight data not related to any other data among all samples.

4. In the validation analysis using PCA, after correcting for mismatched IDS, the ancestry of all the other samples were matched correctly?

A: Yes. Figure 4 showed all the samples that changed races after correcting sample IDs. All the ancestries matched correctly after correcting sample IDs. The other samples not showing here didn’t change their ancestries.
