# Supplementary material for: DRAMS: A tool to detect and re-align mixed-up samples for integrative studies of multi-omics data
Source: PLoS Comput Biol. 2020 Apr 13;16(4):e1007522. doi: 10.1371/journal.pcbi.1007522 (PMC7179940; doi:10.1371/journal.pcbi.1007522)
Supplement: Attachment — Submitted filename: Responce to reviewers.docx [file pcbi.1007522.s020.docx]

Reviewer #1: My comments have been addressed.

Reviewer #2: The authors addressed most of my concerns except the my question #3. It is nice that the authors added a paragraph (lines 276-385) to suggest the use of stringent relatedness score in family data. However, it is still not clear how the relatedness score will affect the result and how to choose the relatedness score in the real data analysis. It is better to test the method using a variety of relatedness score.
A: We appreciate the reviewer for the important suggestion. We used gradient thresholds (range from 0.2 to 0.8) to test the performance of DRAMS using the PsychENCODE BrainGVEX data. According to the results, we found that a loose threshold can lead to a large proportion of overcorrection (S8 Table, Manuscript lines 368-369).
